# JAK3/STAT5 signaling‐triggered upregulation of PIK3CD contributes to gastric carcinoma development

**DOI:** 10.1002/ccs3.12017

**Published:** 2024-02-07

**Authors:** Qingqing Hu, Ning Dou, Qiong Wu, Yong Gao, Yandong Li, Jingde Chen

**Affiliations:** ^1^ Department of Oncology Shanghai East Hospital School of Medicine, Tongji University Shanghai China; ^2^ Department of Radiation Oncology Huadong Hospital Fudan University Shanghai China; ^3^ Department of Oncology Ji'an Hospital Shanghai East Hospital Ji'an China

**Keywords:** gastric cancer, JAK3, PIK3CD, pro‐inflammatory tumor microenvironment, STAT5

## Abstract

Gastric cancer (GC) is one of the most common solid cancers with high incidence and mortality worldwide. Chronic gastritis and consequent inflammatory microenvironment is known as a major cause leading to gastric carcinogenesis. Here we report that PIK3CD that encodes p110δ, a catalytic subunit of the class IA PI3Ks, is overexpressed and tumorigenic in GC and associated with tumor inflammatory microenvironment. By investigating the data from TCGA database and our immunohistochemical staining and quantitative real‐time PCR results from clinical samples, we found PIK3CD exhibits higher expression level in GC tissues compared with adjacent non‐tumorous stomach tissues. Genetic silencing of PIK3CD in GC cells retards proliferation and migration in vitro and tumorigenicity and metastasis in vivo. In contrast, enhanced expression of PIK3CD promotes these phenotypes in vitro. Furthermore, pharmacological inhibition of PIK3CD could reduce GC cell viability and colony formation capacities. More importantly, we reveal a relevant mechanism that PIK3CD, but not PIK3CA and PIK3CB, is transcriptionally regulated by the pro‐inflammatory IL2/JAK3/STAT5 axis and tumor‐infiltrating immune cells such as lymphocytes. These observations may setup a new crosstalk between tumor inflammatory microenvironment, IL2/JAK3/STAT5 signaling and PI3K/AKT signaling. Targeting PIK3CD may be a promising therapy strategy for GC.

## Graphical abstract

The molecular mechanism diagram of contribution of PIK3CD in gastric cancer development. The pro‐inflammatory IL2/JAK3/STAT5 axis upregulates PIK3CD expression by modulating PIK3CD transcription, then PI3K/Akt signaling is activated and promotes gastric carcinoma growth and migration.

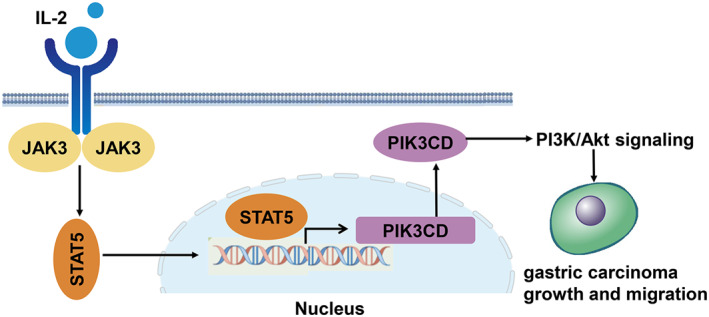

## 1 INTRODUCTION

Gastric cancer (GC) is the fifth most common cancer and the fourth leading cause of cancer death in the world, accounting for approximately 1,080,000 new cases and 760,000 deaths in 2020.^1^ To date, surgical resection remains the main treatment for GC. However, in some cases of GC, local relapses and metastases occur after resection of the primary tumor. In the USA, European countries and China, the overall 5‐year survival rate of GC patients is only about 30%.^2^, ^3^ *Helicobacter pylori* infection is the main cause of GC, contributing to more than 75% of GC cases.^4^ One key role of *H. pylori* infection in GC is the induction of host inflammatory responses. The activation of pro‐inflammatory signaling pathways (such as JAK/STAT, NF‐κB, IL1 and COX2), together with tumor‐infiltrating immune cells (such as lymphocytes and macrophages), contributes to the initiation and progression of GC.^5^, ^6^

The PI3K/Akt signaling axis is one of the most frequently activated pathways in multiple types of human cancer.^7^ PI3Ks belong to a family of lipid kinases, responsible for phosphorylating phosphatidylinositol‐4,5‐bisphosphate (PI‐4,5‐P2) to format phosphatidylinositol‐3 (PIP3). Then PIP3 activates Akt and its downstream effectors, such as oncogenic mTOR signaling.^7^ In mammals, PI3Ks are composed of three classes (class I, II and III), and class I is further divided into subclasses IA and IB.^8^, ^9^ The class IA PI3Ks consist of a regulatory subunit P85 and catalytic subunits p110α, p110β, and p110δ that are encoded by three genes *PIK3CA*, *PIK3CB*, and *PIK3CD*, respectively.^8^, ^9^ In human cancers, *PIK3CA* is frequently mutated, while the other two subunits are generally amplified and overexpressed.^9^ There is accumulating evidence that p110 isoforms of class IA PI3Ks play essential roles in regulating tumor growth, drug‐resistance and angiogenesis.^10^, ^11^, ^12^ Among class IA PI3K p110 isoforms, PIK3CD is mainly expressed in leukocytes and have been widely studied in hematological malignancies.^13^, ^14^, ^15^ Recently, several studies have found that PIK3CD also contributes to cancer progression in several types of solid tumors, such as colorectal cancer (CRC), breast cancer, glioblastoma, squamous cell carcinoma and hepatocellular carcinoma (HCC).^16^, ^17^, ^18^, ^19^, ^20^, ^21^ However, the functions of PIK3CD are poorly investigated and remain unclear in GC.

Here, we aimed to explore the function and underlying mechanism of PIK3CD in the development of GC and found that the expression of PIK3CD is higher in GC tissues than in adjacent normal tissues. PIK3CD upregulation contributes to the proliferation and migration of GC cells in vitro and in vivo. Furthermore, we uncover that PIK3CD overexpression in GC may be induced by the activation of the pro‐inflammatory IL2‐JAK3‐STAT5 signaling axis and PIK3CD may serve as an effective therapeutic target for GC treatment.

## 2 MATERIALS AND METHODS

### 2.1 Patients and tissue specimens

Forty five paired GC tissues and non‐tumor adjacent gastric mucosa samples were collected from Shanghai East Hospital, Tongji University School of Medicine, China. The samples were immediately put into liquid nitrogen and stored at −80°C until used for RNA and protein extraction. A GC tissue microarray (#HStmA180Su15) was purchased from Shanghai Outdo Biotech, which contains 98 cases of patients with GC specimens. Immunohistochemical staining was applied to detect the expression of PIK3CD using an anti‐PIK3CD antibody (#34050, Cell Signaling Technology) at a dilution of 1:50. Staining score was assessed in a blinded manner by two pathologists. On the basis of percentage of positive staining cells and intensity as previously described,^22^ the samples were classified into four categories: Negative, −, <15%; weakly positive, +, 15%–40%; moderate positive, ++, 40%–75% and strongly positive, +++, >75%. All the use of human clinical samples was approved by the Ethics Committee of Shanghai East Hospital.

### 2.2 Cell lines and reagents

Five GC cell lines (SGC7901, AGS, BGC823, MGC803, and HGC27) were obtained from Shanghai Cell Bank of Chinese Academy of Sciences and were routinely maintained in Dulbecco's Modified Eagle Medium (DMEM, Sigma) supplemented with 10% fetal bovine serum (FBS, Gibco) and penicillin/streptomycin (100 units/mL) in a humidified incubator with 5% CO_2_ at 37°C. All cell lines were tested for mycoplasma contamination and mycoplasma‐free cells were used. PI3Kδ inhibitors, including Acalisib (#S5818), compound 7n (#S8693) and Idelalisib (#S2226), STAT3/STAT5 inhibitor SH‐4‐54 (#S7337), STAT3 inhibitor BP‐1‐102 (#S7769), and JAK3 inhibitor JANEX‐1 (#S5903) were purchased from Selleck Chemicals. Human IL‐2 (HY‐P70758) was purchased from MedChemExpress.

### 2.3 RNA interference, overexpression plasmid and cell transfection

Target‐specific small inference RNAs (siRNAs) against PIK3CD and non‐specific sequence (NC), were synthesized and purchased from GenePharma. The sense strand sequences of these siRNAs are following: siPIK3CD‐1 5′‐GCGCCAAGAUGUGCCAAUUdTdT‐3′; siPIK3CD‐2 5′‐CCACAGGUGAUCCUAACAUdTdT‐3′; siNC 5′‐UUCUCCGAACGUGUCACGUdTdT‐3′. To overexpress PIK3CD and STAT5, the expression plasmids, pHAGE‐PIK3CD (Addgene, #116773)^23^ was a gift from Gordon Mills & Kenneth Scott. pEnter‐STAT5A (Accession Number: NM_003152) and pEnter‐STAT3 (Accession Number: NM_003150) were purchased from Shandong Vigene Biosciences, China. Cell transient transfection with above siRNAs against PIK3CD or plasmids was performed with Lipofectamine 3000 (Invitrogen) according to the manufacturer's instructions. GC cells were transduced with lentiviral particles knocking down PIK3CD or control (LVshPIK3CD‐1, LVshPIK3CD‐2 or LV‐shNC). These lentiviral particles were packaged from GenePharma, Shanghai using corresponding siPIK3CD‐1, siPIK3CD‐2 and siNC sequence. Stably transfected cell lines were isolated for 2 weeks in the presence of puromycin.

### 2.4 Western blot analysis

GC tissues or GC cells were lyzed in RIPA buffer for 30 min on ice supplemented with a protease inhibitor cocktail, followed by centrifugation at 12,000 rpm for 10 min. The lysates was then diluted in 5 × SDS loading buffer and boiled for 3 min. The protein mixtures were separated by sodium dodecyl sulfate polyacrylamide gel electrophoresis and electro‐blotted to a PVDF membrane (EMD Millipore). After being blocked with non‐fat milk for about 1 h at room temperature, the membrane was then incubated 2 h with the primary antibodies at room temperature or overnight at 4°C, followed by the secondary antibody for another 1 h. The protein detection was performed with Odyssey Infared Imaging System (Li‐COR). Primary antibodies used in this study as follows: actin (1:500, Santa Cruz Biotechnology, #8432), PI3 Kinase p110α (1:1000, Cell Signaling Technology, #4249), PI3 Kinase p110 δ (1:1000, Cell Signaling Technology, #34050), PI3 Kinase p110β (1:1000, Cell Signaling Technology, #3011), STAT5 (1:1000, Cell Signaling Technology, #25656), JAK3 (1:1000, Cell Signaling Technology, #8827), Phospho‐Akt (Ser473) (1:1000, Cell Signaling Technology, #9271), Akt Antibody (1:1000, Cell Signaling Technology, #9272).

### 2.5 RNA extraction and qRT‐PCR

Total RNA was isolated from GC cell lines or GC tissues with RNAiso Plus reagent (TaKaRa, Japan) based on the manufacturer's protocol. The PrimeScriptTM RT Reagent Kit with gDNA Eraser, obtained from TaKaRa, Japan, was used to synthesize the first strand cDNA. Quantitative real‐time PCR (qRT‐PCR) was performed with the SYBR green reagent (TaKaRa, Japan) on ABI QuantStudioTM 6 Flex system (Thermo Fisher Scientific). Relative quantification of PCR products was performed with the comparative Ct method (2^−ΔΔCt^), and β‐actin was used an endogenous control. The results were all repeated three times. The following primer pairs were used to amplify and measure the amount of PIK3CD, 5′‐CATATGTGCTGGGCATTGGC‐3′ and 5′‐TTTCACAGTAGCCCCGGAAC‐3′). The primers for β‐catenin: 5′‐ CCTGGCACCCAGCACAATG‐3′ and 5′‐ GGGCCGGACTCGTCATACT‐3′).

### 2.6 Cell viability assays

Twenty four h after transfection, 3000 GC cells/well were cultivated in fresh 96‐well plates in triplicate and cultured in normal medium for 5–7 days. According to the instructions of the manufacturer, 10 μL of CCK8 reagent (Cell Counting Kit‐8, Dojindo) was added into each well and incubated for 1 h 15 min at 37°C. Absorbance at 450 nm was measured in an automated plate reader. These experiments were repeated at least three times.

### 2.7 Colony formation assays

Stably infected GC cells were seeded in 6‐well culture plates at 2 × 10^3^ per well. After 14 days, the cell colonies were stained with crystal violet (0.5%), counted and photographed. Each experiment was independently repeated at least three times.

### 2.8 Wound healing assays

The stably expressed shPIK3CD or control GC cells were plated into six‐well plates. When cell density reached about 90%, a straight scratch was made at the bottom of each well with a plastic tip. Cell debris was then removed with PBS, and FBS‐free DMEM was added. After cultured for 24 or 48 h, cell images were taken at indicated time point by an inverted microscope (Nikon). The experiment was repeated three times.

### 2.9 Animal experiments

Five‐week‐old BALB/c nude mice (18–20 g) were purchased from SLAC Laboratories Animal, Shanghai, China. 2 × 10^6^ of GC cells infected with LVshPIK3CD‐1, LVshPIK3CD‐2 or LV‐shNC were subcutaneously injected into the dorsal flank of these mice (*n* = 6 or 7 each group), respectively. The weight of formed xenografts was measured after 4 weeks. For the lung metastasis assay, 1 × 10^6^ of SGC7901 cells (LV‐shNC, LV‐shPIK3CD‐1 and LV‐shPIK3CD‐2, *n* = 5 each group) were injected into the tail vein of nude mice. After 30 days, all mice were sacrificed. The lung metastatic tumors were calculated. A part of lung tissues were fixed in 4% paraformaldehyde, embedded in paraffin, stained with hematoxylin and eosin. Animal experiments were performed under protocols approved by the Animal Care and Use Committee of Shanghai East Hospital.

### 2.10 Dual luciferase reporter assay

A 923‐bp promoter sequence of PIK3CD including the predicted binding site for STAT5 was cloned into pGL3‐basic vector (Promega) to generate wild‐type (WT) reporter plasmid PIK3CD‐WT, and then the predicted binding site of STAT5 were mutated by site‐directed mutagenesis (MUT). For the luciferase reporter assays, MGC803 and AGS cells were seeded into 24‐well plates and incubated for 24 h. The plasmids (WT‐PIK3CD or MUT‐PIK3CD, plus pRL‐SV40 Renilla luciferase construct) were co‐transfected with STAT5 or control vector into MGC803 and AGS cells using Lipofectamine 3000 (Invitrogen). After incubation for 24 h, cells ere lyzed and the Dual‐Luciferase Reporter Assay system (Promega) was used to monitor the relative luciferase activity.

### 2.11 ChIP assay

AGS cells were used to performed chromatin immunoprecipitation (ChIP) assays with an EZ ChIP Kit (Merk Millipore) in accordance with the manufacturer's instructions. The STAT5 antibody (Cell Signaling Technology, #25656) and control IgG immunoprecipitated DNA from AGS cells was amplified for the promoter region (from −624 to −355) of PIK3CD via PCR and ran on a 2% agarose gel. The following primers were used in this assay: Forward: 5′‐CCAAATTGCTGGGATTGCAG‐3′; Reverse: 5′‐GTAACCTGCAGCTCTAGGCA‐3′.

### 2.12 Statistical analysis

Each in vitro experiment was repeated at least three times. In vitro and in vivo data were shown as mean ± standard deviation and analyzed using GraphPad Prism 7.0 software. The significance of differences between groups was assessed by Student's *t*‐test or one‐way analysis of variance. *χ* ^*2*^ test was used to compare the difference of positive rate of PIK3CD in cancer and non‐cancer groups. *p* values < 0.05 were considered statistically significant. *p < 0.05, **p < 0.01.

## 3 RESULTS

### 3.1 PIK3CD was frequently upregulated in GC

**FIGURE 1:**
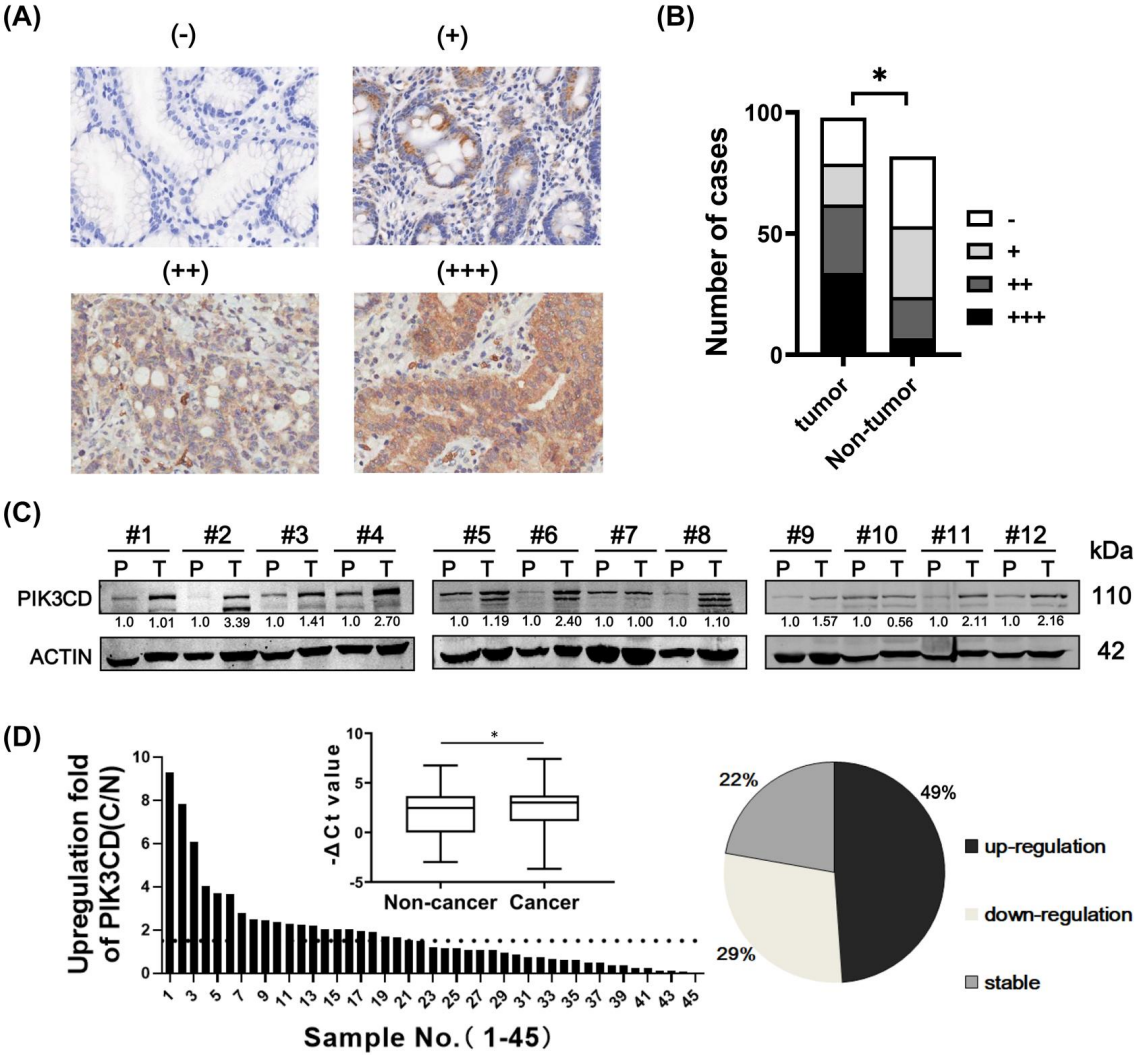
The expression pattern of PIK3CD in GC specimens. (A) Representative immunohistochemical staining images of PIK3CD on a GC tissue microarray were shown. (−) represents negative; (+) represents weak positive; (++) represents moderate positive; (+++) represents strong positive. Of these representative pictures, (−) came from normal stomach tissues, (+) came from cancer‐adjacent tissues, and (++)/(+++) came from gastric cancer tissues. Scale bar: 50 μm. (B) The positive ratio of PIK3CD staining in the GC tissue microarray. *n* = 98, for GC tissues; *n* = 82, for non‐tumor tissues. *p < 0.05. (C) The protein expression of PIK3CD in 12 pairs of GC samples was analyzed by western blotting. P, paratumor tissues; T, tumor tissues. (D) The mRNA level of PIK3CD in 45 pairs of GC tissues and adjacent non‐cancer tissues were determined by qRT‐PCR. The percentage of PIK3CD expression alteration was shown. Upregulation: C/N ≥ 1.5; Downregulation: C/N ≤ 0.67; Stable: >0.67 but <1.5. GC, gastric cancer.

To explore the expression of PIK3CD in human GC, a GC tissue microarray containing 98 cases of GC clinical samples and 83 cases of adjacent normal stomach tissues was firstly measured by immunohistochemical staining with a PIK3CD‐specific antibody. Representative images were shown in Figure 1A and the results showed an upregulated PIK3CD expression in GC compared with non‐tumor tissues (Figure 1B). Regretfully, we have also evaluated the correlation between PIK3CD expression and tumor grade or metastasis of these GC patients, but there is no significance to be found. In addition, we also evaluated the mRNA and protein level of PIK3CD in additional 45 paired human GC samples and adjacent normal tissues. In accordance with TCGA database analyzed by GEPIA (http://gepia.cancer‐pku.cn/), a web server for analyzing RNAseq data based on TCGA (Figure S1a), the expression of PIK3CD is significantly higher in GC tissues than in adjacent normal tissues (Figure 1C,D). Furthermore, survival analysis of GC patients from Kaplan‐Meier Plotter online tools (http://kmplot.com/) revealed that high expression of PIK3CD is significantly associated with poor outcomes of GC patients (HR = 1.52, *p* = 1.2e‐06) (Figure S1b). These results suggested that PIK3CD is overexpressed in GC and may play an oncogenic role in GC development.

### 3.2 Knockdown of PIK3CD inhibited GC cell proliferation and migration in vitro

**FIGURE 2:**
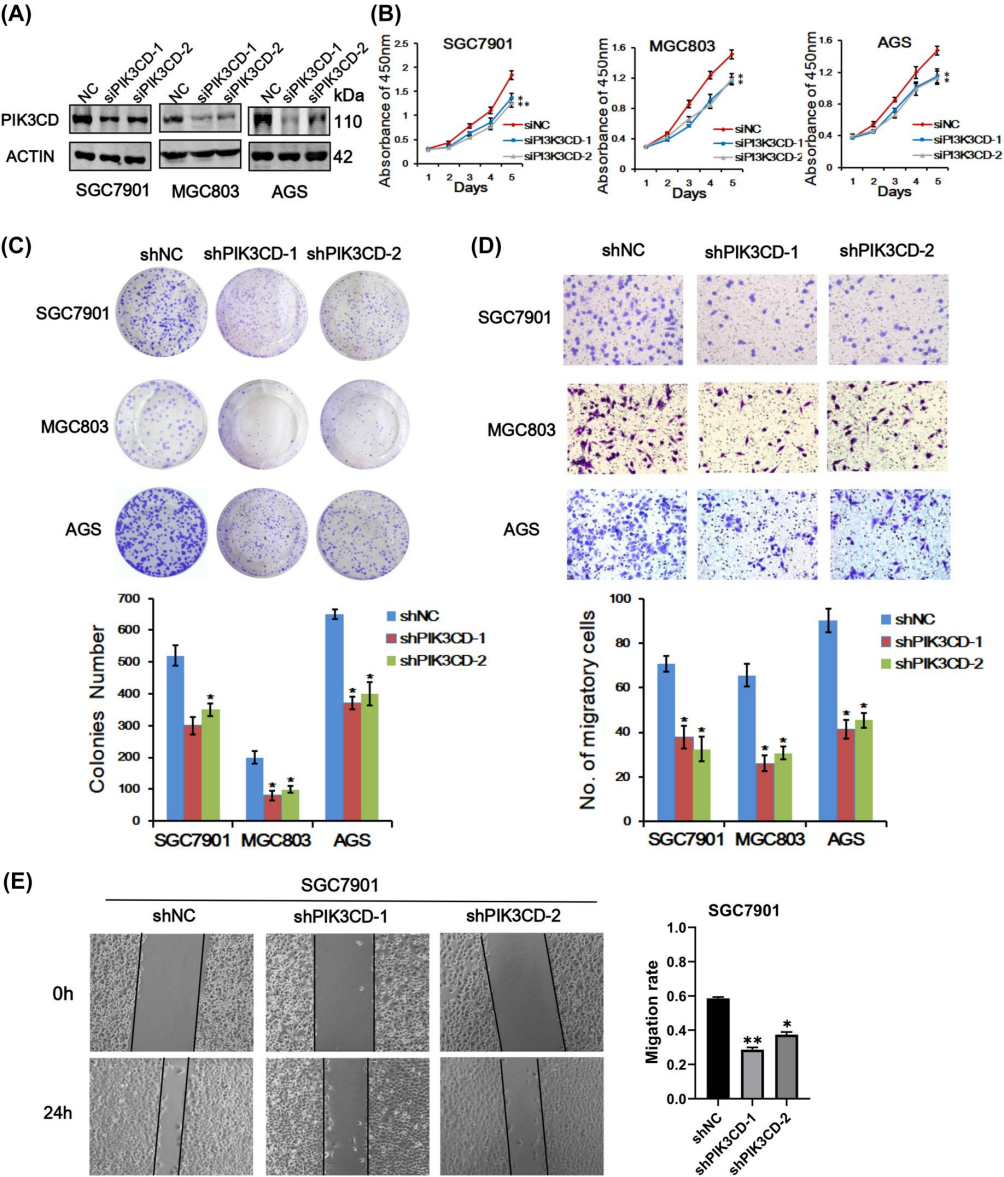
PIK3CD silencing attenuated GC cell growth and migration capacities. (A) PIK3CD knockdown efficiency was detected by western blotting in SGC7901, MGC803 and AGS cells. (B) CCK‐8 analyses of PIK3CD knockdown in three cell lines as indicated. (C) Colony formation assays of PIK3CD stable knockdown in SGC7901, MGC803 and AGS cells. (D) Cell migration ability was determined by Transwell assays in PIK3CD stable knockdown cells and control cells as indicated. (E) Wound healing experiments were performed to evaluate the motility ability of SGC7901 cells in which PIK3CD was stably silenced. All the data above were expressed as mean ± SD, *p < 0.05, **p < 0.01. GC, gastric cancer; SD, standard deviation.

To determine whether PIK3CD plays a role on the growth and migration of GC cells, we firstly silenced PIK3CD expression by two siRNAs which were chemically synthesized and transiently transfected into SGC7901, MGC803 and AGS cells, respectively. As shown in Figure 2A, the expression of PIK3CD was significantly downregulated after siRNA transfection. CCK8 method was then employed and the cell viability results exhibited that PIK3CD knockdown evidently suppressed the proliferation of these three cell lines (Figure 2B). Furthermore, lentivirus‐mediated stable knockdown of PIK3CD in SGC7901, MGC803 and AGS cells also led to fewer and smaller colonies in culture plates in comparison with corresponding control cells (Figure 2C). Meanwhile, the migration ability of GC cells with stable knockdown of PIK3CD was significantly attenuated as demonstrated in transwell assays and wound healing assays (Figure 2D,E). These collective results suggested that PIK3CD plays a key role in promoting cell proliferation and migration in GC.

### 3.3 Overexpression of PIK3CD significantly promoted GC cell proliferation and migration

**FIGURE 3:**
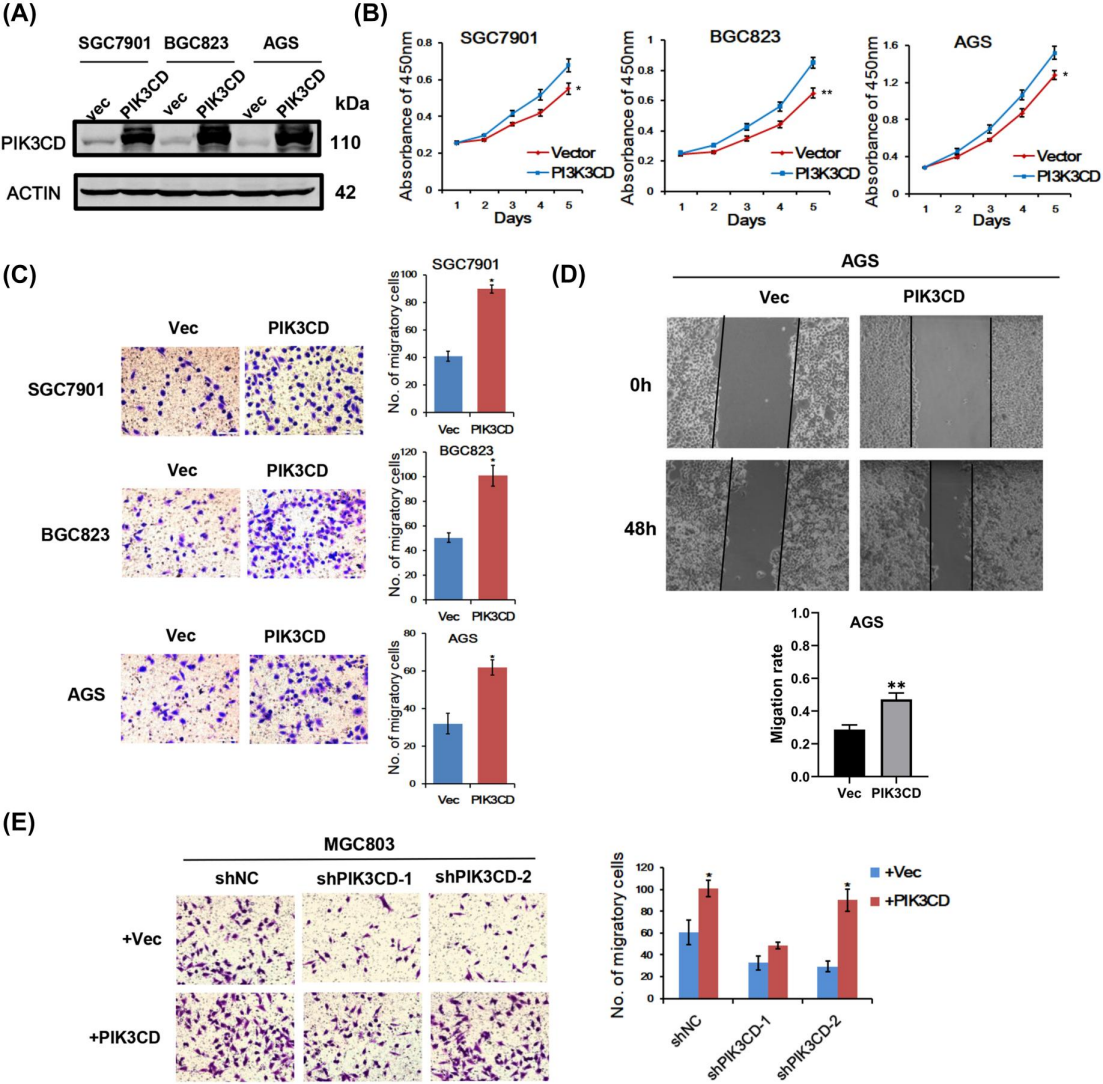
The effect of ectopic expression of PIK3CD on GC cell proliferation and migration. (A) Western blot results of PIK3CD overexpression in SGC7901, BGC823 and AGS cells via transient transfection with pEnter‐PIK3CD and control vector. (B) The growth rates of indicated cells with or without PIK3CD ectopic expression were examined by CCK‐8 assays. (C) Transwell chamber assays were used to detect cell migration affected by PIK3CD overexpression in three cell lines as indicated. (D) The effect of PIK3CD expression on wound healing capacity of AGS cells. (E) Ectopic expression of PIK3CD rescued the migratory defect induced by PIK3CD stable knockdown in MGC803 cells. *p < 0.05, **p < 0.01.

To further estimate the functions of PIK3CD, we overexpressed PIK3CD in GC cells by plasmid transfection (Figure 3A). The results of CCK8 assays demonstrated that PIK3CD overexpression enhanced the proliferation of SGC7901, BGC823 and AGS cells (Figure 3B). Through transwell assay and wound healing assay, we also found that PIK3CD overexpression significantly promoted cell migration in these GC cells (Figure 3C,D). Importantly, the results of transwell assays showed that transfection with the PIK3CD‐overexpressed plasmid in GC cells could restore the effects of PIK3CD knockdown in shPIK3CD‐2 cells, but not in shPIK3CD‐1 cells (Figure 3E). That may be because shPIK3CD‐1 targets sequence within PIK3CD coding region and shPIK3CD‐2 targets sequence out of PIK3CD coding region. Collectively, these results clarified that PIK3CD overexpression may play oncogenic roles in GC.

### 3.4 Downregulation of PIK3CD inhibited tumor growth and metastasis of GC in vivo

**FIGURE 4:**
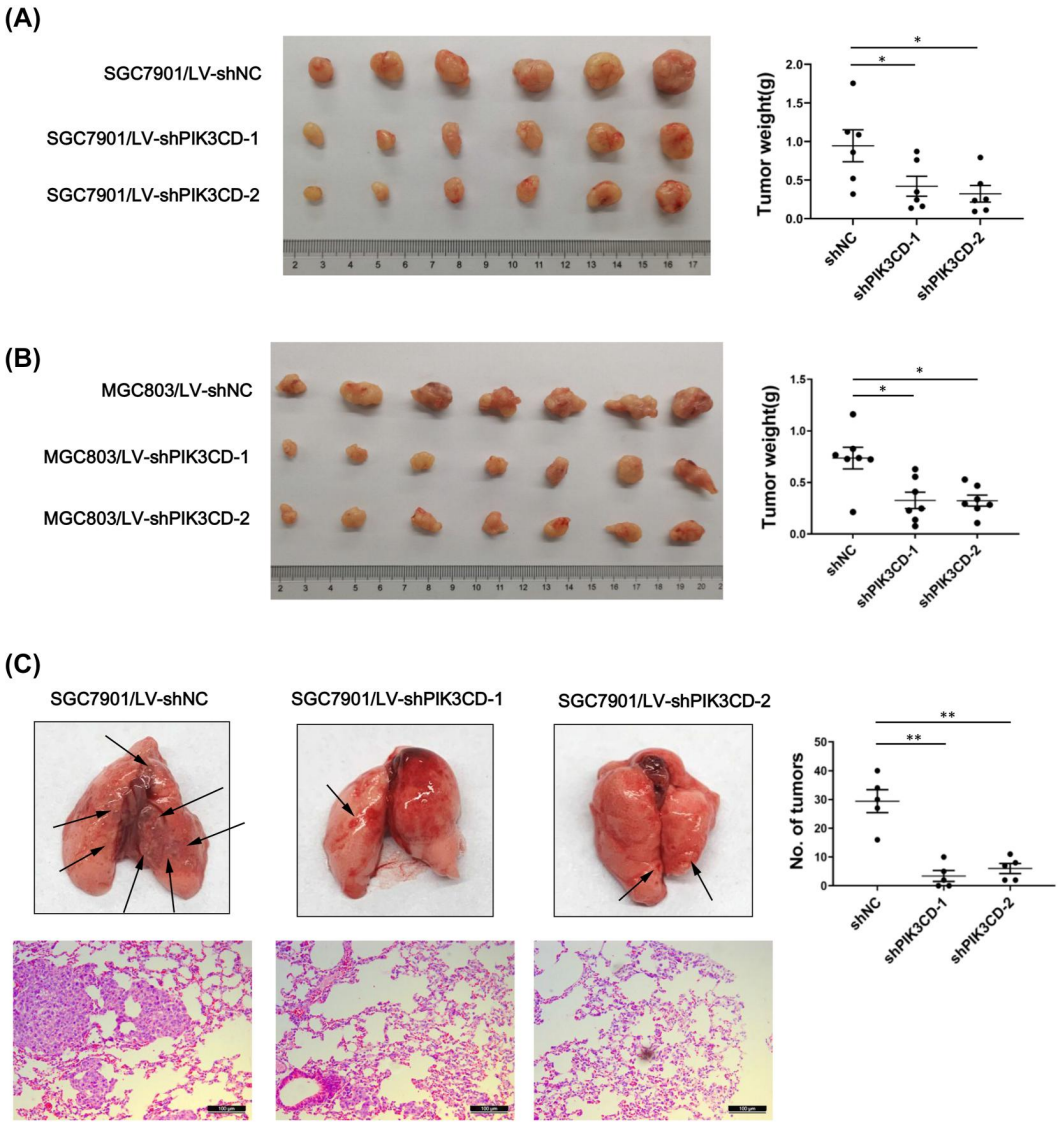
PIK3CD knockdown reduced tumorigenicity of GC cells in vivo. (A, B) PIK3CD stable knockdown in SGC7901 or MGC803 cells inhibited subcutaneous xenograft tumor growth. The tumors formed in nude mice were shown. Tumor weight was measured after excision. (C) Lung metastasis assay was performed with SGC7901 cells as indicated by tain vein injection. Representative images of lung metastasis and H&E staining were shown. Scale bar: 100 μm. All of above values are expressed as mean ± SD, *p < 0.05, **p < 0.01. GC, gastric cancer; H&E, hematoxylin and eosin; SD, standard deviation.

To investigate the effect of PIK3CD on tumorigenicity in vivo, we subcutaneously transplanted shPIK3CD‐1, shPIK3CD‐2 and shNC GC cells into nude mice. As concluded from the observation of removed tumors, we found that tumor weight of the PIK3CD knockdown group was significantly lower than that in the control group (Figure 4A,B). Meanwhile, mouse pulmonary metastasis model indicated that the tumors on the lung surface formed from PIK3CD knockdown cells were significantly less than those of control cells (Figure 4C). These results demonstrated that PIK3CD knockdown suppresses GC cell growth and metastasis in mouse models.

### 3.5 PIK3CD inhibitors suppressed the growth of GC cell lines in vitro

**FIGURE 5:**
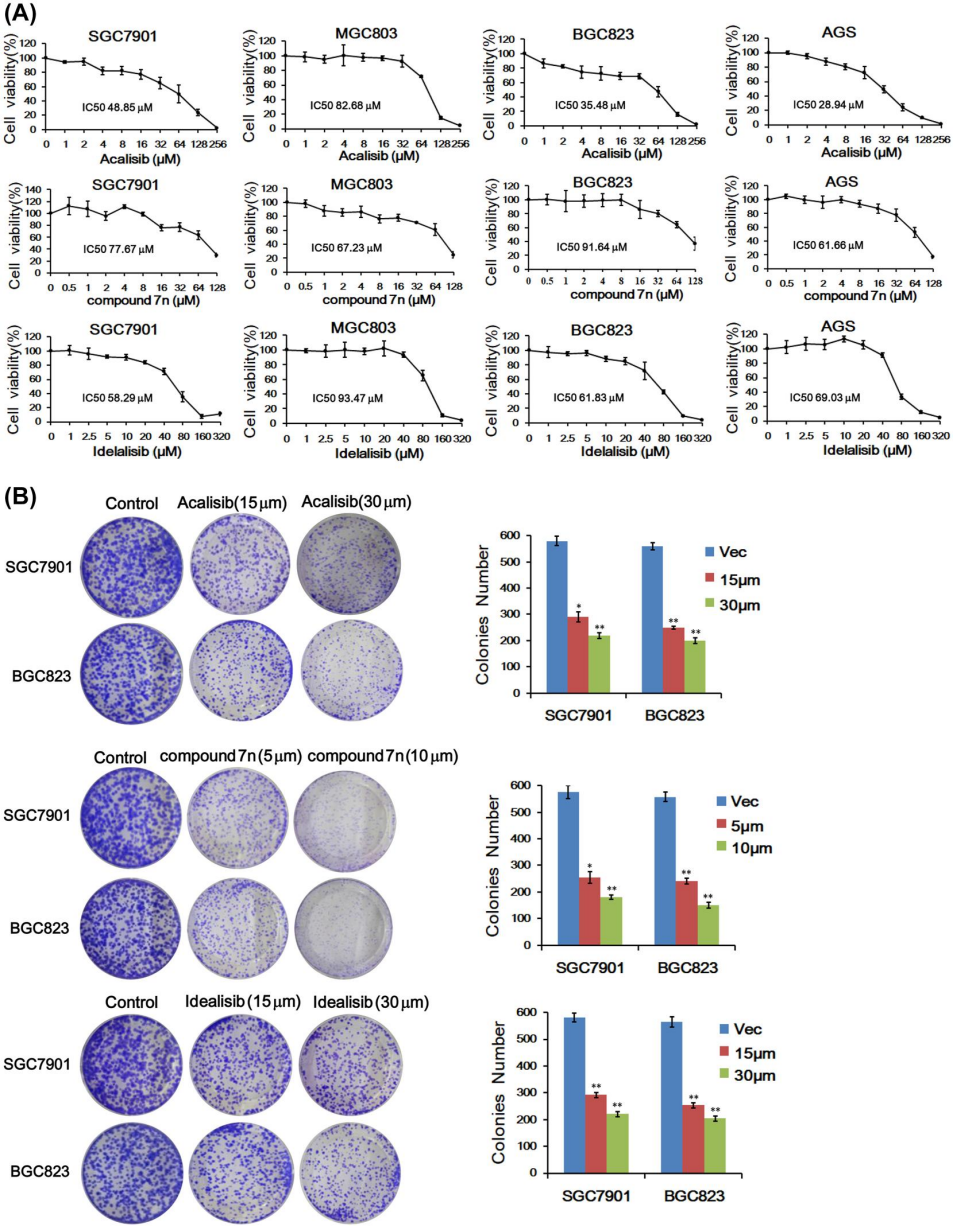
PIK3CD inhibitors suppressed GC cell viability in vitro. (A) Four GC cell lines as indicated were treated with Acalisib, Compound 7n and Idelalisib for 48 h, and then CCK‐8 analysis was performed. IC_50s_ were determined by GraphPad Prism 7 using the nonlinear regression subprogram. (B) Colony formation assays with GC cells treated with different concentrations of Acalisib, Compound 7n and Idelalisib for 2 weeks. Data are represented as mean ± SD of independent experiments (*n* = 3). *p < 0.05, **p < 0.01. GC, gastric cancer; IC_50s_, inhibitory concentrations; SD, standard deviation.

Subsequently, we examined the viability and proliferative abilities of the four GC cell lines (SGC7901, SGC803, BGC823 and AGS) in response to different PI3K inhibitors, including acalisib, compound 7n and idelalisib, at a series of concentrations. Through CCK‐8 assay, the half‐maximal growth inhibitory concentration (IC_50_) of these inhibitors on different GC cell lines was calculated. As a result, the viability and proliferation of GC cells were suppressed by all three PI3K inhibitors in a dose‐dependent manner (Figure 5A). Among these inhibitors, acalisib showed more potent effects than two others (compound 7n and idelalisib), though the MGC803 cell line was more sensitive to compound 7n (Figure 5A). We then further examined the function of PI3K inhibitors on the proliferative ability of SGC7901 and BGC823 cell lines by colony formation assays. Cultured GC cells were treated with two doses (15 and 30 μM) for acalisib and idelalisib, and two doses (5 and 10 μM) for compound 7n, respectively. DMSO treatment served as respective negative control groups. The results showed that PI3Kδ inhibitors significantly reduced the colonies number of SGC7901 or BGC823 cells (Figure 5B). In summary, these data indicated that inhibition of PI3Kδ isoform by acalisib, compound 7n and idelalisib has potent inhibitory effects on the growth of GC cells.

### 3.6 JAK3/STAT5 signaling activates PIK3CD expression in GC

**FIGURE 6:**
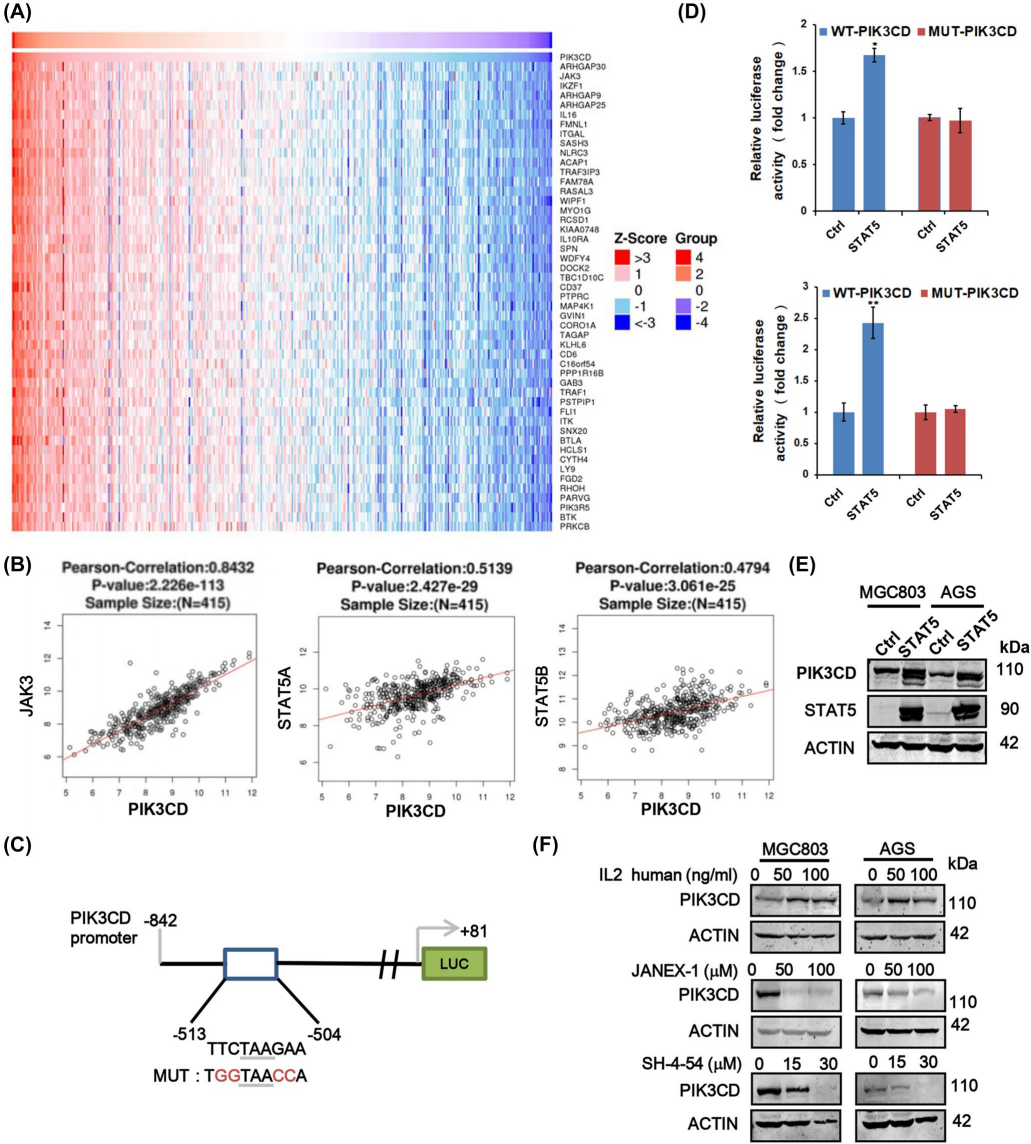
PIK3CD was positively regulated by JAK3/STAT5 signaling. (A) Heat maps of top 50 genes positively correlated with PIK3CD expression in STAD cohort of TCGA by LinkedOmics online tools. STAD, stomach adenocarcinoma. (B) The expression of PIK3CD was positively associated with JAK3, STAT5A and STAT5B in TCGA by Pearson correlation analysis. (C) The potential binding site of STAT5 in the promoter of PIK3CD. The four red nucleotides were mutated from wild‐type binding sites for construction of dual luciferase reporter plasmid (MUT). (D) The relative activities of PIK3CD promoter were examined by dual luciferase reporter kit in GC cells with STAT5A overexpression. *p < 0.05, **p < 0.01. (E) The protein expression was tested by western blotting upon STAT5A overexpression in MGC803 and AGS cells. (F) Cells were treated with human IL‐2, JANEX‐1 and STAT5 inhibitor (SH‐4‐54) as indicated concentrations. PIK3CD expression was examined by western blot analysis. JANEX‐1, JAK3 inhibitor.

To explore the relationship of PIK3CD and other genes/signaling pathways in GC, we performed correlation analyses of 415 GC samples from TCGA database using LinkedOmics (http://www.linkedomics.org/login.php). Notably, we found that the expression of PIK3CD is positively associated with JAK3 (*R* = 0.8432, *p* < 0.05), a kind of Janus tyrosine kinase that has been wildly reported to promote cancer progression^24^, ^25^, ^26^, ^27^ (Figure 6A). Importantly, the expression of PIK3CD is also positively associated with the expression levels of STAT5A (*R* = 0.5139, *p* < 0.05) and STAT5B (*R* = 0.4794, *p* < 0.05), two downstream genes of JAK3 signaling (Figure 6B). Moreover, GEPIA (http://gepia.cancer‐pku.cn/) was applied to confirm that PIK3CD was positively associated with the signatures of JAK3/STAT5 signaling pathway, which consists of 96 related genes collected from the KEGG database (Figure S2a). By searching the consensus of STAT5 binding sites (5′‐TTCNNNGAA‐3′) in the promoter of PIK3CD, we found a putative binding site of STAT5 at −513 to −504 bp (Figure 6C). To examine whether STAT5 can regulate PIK3CD transcription via this putative site, we cloned the linear sequence of PIK3CD promoter (PIK3CD‐WT) as well as its mutant form with the potential STAT5‐binding site mutation (PIK3CD‐MUT) into luciferase reporter vectors, which were then transfected into MGC803 and AGS cells with STAT5A overexpression plasmid or control vector. The relative luciferase activities were measured and the data indicated that STAT5A significantly enhanced the luciferase activities of WT promoter of PIK3CD, but not the mutant promoter (Figure 6D). Importantly, a 274 bp fragment containing the STAT5 binding site could be co‐immunoprecipitated with endogenous STAT5 protein (Figure S2b). These results suggested that STAT5 may transcriptionally activate PIK3CD in GC cells. On the other hand, overexpression of STAT5A also increased the protein and mRNA levels of PIK3CD as demonstrated in Figure 6E and Figure S2c. In addition, MGC803 and AGS cells were treated with human IL‐2 (interleukin‐2), JANEX‐1 (JAK3 inhibitor), SH‐4‐54 (STAT3/STAT5 inhibitor) and then performed western blot and qRT‐PCR assays. The results showed that IL‐2 markedly led to the upregulation of PIK3CD, while JANEX‐1 and SH‐4‐54 evidently downregulated the expression of PIK3CD (Figure 6F and Figure S2d). Taken together, these results demonstrated that PIK3CD overexpression in GC cells is partially induced by IL2/JAK3/STAT5 signaling axis.

### 3.7 The impact of tumor infiltrating immune cells, inflammatory response and JAK3/STAT5 signaling on PIK3CA, PIK3CB and PIK3CD in GC

**FIGURE 7:**
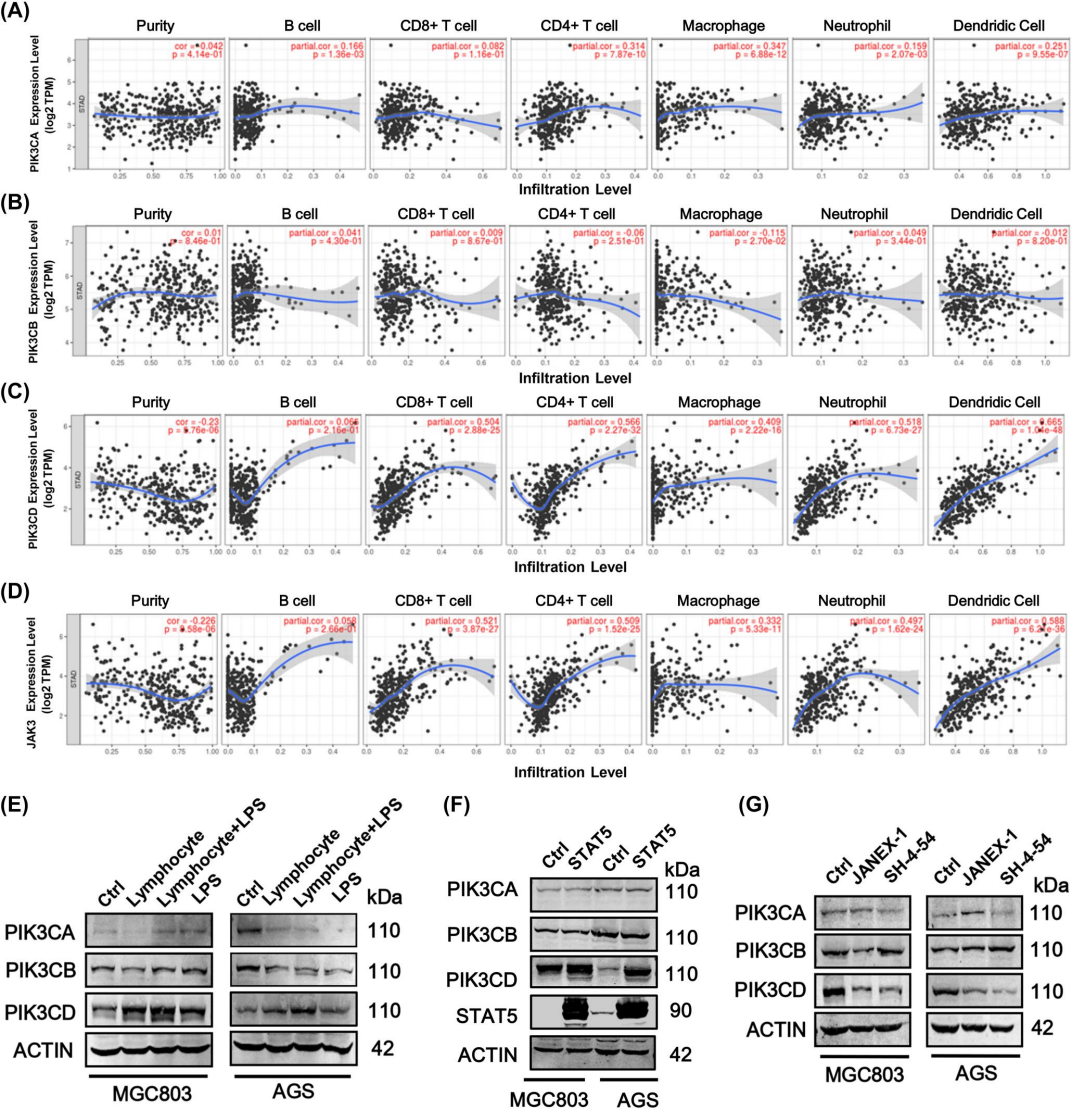
The correlations between PI3K subunits, JAK3 and immune cell infiltration in GC. (A) PIK3CA and immune cell infiltration. The correlation analysis between each gene with immune cell infiltration was performed via TIMER algorithm. (B) PIK3CB and immune cell infiltration. The correlation analysis between each gene with immune cell infiltration was performed via TIMER algorithm. (C) PIK3CD and immune cell infiltration. The correlation analysis between each gene with immune cell infiltration was performed via TIMER algorithm. (D) JAK3 and immune cell infiltration. The correlation analysis between each gene with immune cell infiltration was performed via TIMER algorithm. (E) Pro‐inflammatory lymphocyte and LPS had significant impact on PIK3CD expression, but not for PIK3CA and PIK3CB. (F) The effect of overexpression of STAT5 on protein expression of PIK3CA, PIK3CB and PIK3CD. (G) The effect of JANEX‐1 and STAT5 inhibitor (SH‐45‐4) on protein expression of PIK3CA, PIK3CB and PIK3CD. GC, gastric cancer; JANEX‐1, JAK3 inhibitor; LPS, lipopolysaccharide.

TIMER algorithm was employed to analyze the relationship between JAK3, PIK3CA, PIK3CB, PIK3CD and tumor‐infiltrating immune cells in stomach adenocarcinoma (https://cistrome.shinyapps.io/timer/). As a result, PIK3CA expression has positive correlations with infiltrating levels of macrophages, CD4^+^ T cells, and dendritic cells (*R* > 0.2, *p* < 0.05) (Figure 7A) and PIK3CB expression has no marked correlations with infiltrating levels of any types of immune cells (Figure 7B). Interestingly, we found that the expression of PIK3CD and JAK3 has significant positive correlations with infiltrating levels of CD8^+^ T cells, CD4^+^ T cells, macrophages, neutrophils and dendritic cells (*R* > 0.2, *p* < 0.05) (Figure 7C,D). These data implied that PIK3CD has more close relationship with tumor‐infiltrating immune cells or immune response in tumor microenvironment.

To verify this hypothesis, we treated MGC803 and AGS cells with inflammatory lymphocyte and LPS (lipopolysaccharide) to induce an inflammatory response. As expected, lymphocyte, LPS and the combination of lymphocyte and LPS significantly increased the expression of PIK3CD and no effect on the expression of PIK3CA and PIK3CB (Figure 7E). Subsequently, we further explored the relationships between PIK3CD and pro‐inflammatory JAK3/STAT5 signaling pathways. As shown in Figure 7F, overexpression of STAT5 significantly upregulated the expression of PIK3CD in MGC803 cells, whereas the expression of PIK3CA and PIK3CB expression didn't change. Consistently, inhibition of JAK3/STAT5A signaling through JANEX‐1 (JAK3 inhibitor) and SH‐4‐54 (STAT5 inhibitor) could only decrease the expression of PIK3CD in both MGC803 and AGS cells (Figure 7G). These results above suggested that of the three PI3K p110 isoforms, PIK3CD has more close correlation with inflammatory microenvironment of gastric carcinoma.

## 4 DISCUSSION

Class IA PI3K p110 isoforms are generally dysregulated in human cancers. For PIK3CD, parts of its functions and relevant mechanisms have been revealed in some types of solid cancer.^28^ In CRC, PIK3CD is found to promote tumor cell proliferation and migration through activating AKT/GSK‐3β/β‐catenin axis.^17^ In HCC, PIK3CD/AKT/GSK3β axis contributes to immune escape by increasing PD‐L1‐induced CD8^+^ T‐cell exhaustion.^29^ In glioblastoma, PIK3CD plays its oncogenic role via activation of cytoskeletal proteins PAK3 and PLEK2.^21^

In the present study, we have demonstrated the important oncogenic roles of PIK3CD in GC for the first time. Upregulation of PIK3CD contributes to GC cell growth and metastasis both in vivo and in vitro, consistent with its roles in other cancer types reported previously. Mechanistically, we found that PIK3CD is transcriptionally activated in GC by the IL2/JAK3/STAT5 axis and then triggers downstream AKT signaling pathway. Notably, the IL2/JAK3/STAT5 axis has been reported critical for inflammatory responses, and plays key roles in immune‐related pathological processes in cancer, such as contributing to cancer‐related pain, tumor cell survival and T‐cell exhaustion.^30^, ^31^, ^32^, ^33^, ^34^ In addition to cascading with pro‐inflammatory signaling pathways, PIK3CD overexpression is also closely associated with the activation of tumor‐infiltrating immune cells such as CD8^+^ and CD4^+^ T lymphocytes, which is analyzed by TIMER algorithm. Co‐culture GC cells with human lymphocytes significantly increased the expression of PIK3CD, further confirming our conclusions.

Although all class IA PI3K p110 isoforms may have important functions in human cancers via AKT activation,^35^, ^36^, ^37^ in our study we found that only PIK3CD (p110δ) expression is induced by pro‐inflammatory responses and immune infiltration cells. These findings highlight the unique roles of PIK3CD in response to immune microenvironment of GC. As known, most GC patients share a non‐resolved inflammatory condition, owing to a chronic gastritis caused by *H. pylori* infection.^38^, ^39^, ^40^, ^41^ In a paracrine or autocrine manner, GC cells receive tumorigenic signals from pro‐inflammatory cells and cytokines, which boost tumor initiation and progression.^42^, ^43^, ^44^ In this regard, PIK3CD may be a candidate factor involved in inflammation to cancer transformation.

So far, two PI3Kδ‐specific inhibitors have been approved by the FDA for the treatment of hematological malignances^45^: Idelalisib (CAL‐101, specific for PI3Kδ) and duvelisib (IPI‐145, specific for PI3Kδ and γ).^45^ One major obstacle in the clinical progression of PI3Kδ inhibitors is the management of drug‐related toxicities in cancer patients.^46^ The side effects related to p110δ subunits are generally myelosuppression, transaminitis and gastrointestinal.^46^, ^47^, ^48^ In B‐cell malignancy, isoform‐specific PI3Kδ inhibitors exhibit better effects compared to pan or dual PI3K inhibitors due to its narrower toxicity profile.^49^ However, the efficacy and toxicity of PI3Kδ inhibitors need to be estimated in solid tumors,^50^ including GC. Through using PI3Kδ inhibitors acalisib, compound 7n and idelalisib, we confirm that pharmacological inhibitions of PI3Kδ restrain GC cell growth in vitro. The limitation of the current study includes lack of animal experiments for PI3Kδ inhibitors. Especially, it is difficult to find an available mouse GC cell line for evaluating the efficacy of PI3CD inhibitors on inflammatory microenvironment in mouse models.

In conclusion, PIK3CD overexpression cascades with the activation of pro‐inflammatory IL2‐JAK3‐STAT5 axis and tumor‐infiltrating immune cells, contributing to GC development. The activation of PIK3CD is critical for the proliferation and migration of GC cells in vitro and in vivo. Future studies should further investigate the mechanisms by which PIK3CD regulate GC progression and estimate the effects of PI3Kδ inhibitors in pre‐clinical animal models of GC.

## AUTHOR CONTRIBUTIONS

Qingqing Hu and Ning Dou performed cellular and animal experiments and analyzed data. Qingqing Hu and Qiong Wu carried out molecular experimental and clinical analysis. Yong Gao, Jingde Chen and Yandong Li supervised this research, including experimental design and data interpretation. Qingqing Hu and Yandong Li wrote the manuscript.

## CONFLICT OF INTEREST STATEMENT

The authors have declared that they have no competing interests.

## ETHICS STATEMENT

Studies involving humans were approved by the Ethical Committee of Shanghai East Hospital and animal experiments were performed under protocols approved by the Animal Care and Use Committee of Shanghai East Hospital, Tongji University.

## Supporting information

Supporting Information S1

Figure S1

Figure S2

## Data Availability

All relevant data in this study are included in this published article and its supplementary files.
