# Supplementary material for: JAK3/STAT5 signaling‐triggered upregulation of PIK3CD contributes to gastric carcinoma development
Source: J Cell Commun Signal. 2024 Feb 7;18(1):e12017. doi: 10.1002/ccs3.12017 (PMC10964935; doi:10.1002/ccs3.12017)
Supplement: Supplementary file 1 — Supporting Information S1 [file CCS3-18-e12017-s001.docx]

**Supplementary Figure 1. PIK3CD is overexpressed in GC and associated with poor outcomes of GC patients. (a) Differential expression of PIK3CD in normal tissues and primary gastric cancers in TCGA-STAD cohort (Stomach adenocarcinoma) analyzed by GEPIA online tool. (b) Kaplan-Meier overall survival plot comparing patients with high PIK3CD expression (red line; n = 435) and low PIK3CD expression in tumors (black line; n = 440; P < 0.001, Log-rank test).** The high/low groups were determined by median value.

**Supplementary Figure 2. PIK3CD is regulated by IL2/JAK3/STAT5 signaling at mRNA level. (a) The expression correlation between PIK3CD and JAK3/STAT5 signaling pathway signature analyzed with GEPIA tools. (b) The immunoprecipitated promoter fragment of PIK3CD by anti-STAT5 antibody was analyzed via PCR and agarose gel electrophoresis. (c) qRT-PCR was employed to detect the PIK3CD expression upon STAT5A overexpression in GC cells. (d) qRT-PCR results were shown when GC cells were treated with IL-2, JANEX-1 and SH-45-4.** *p<0.05, **<0.01.
