# Supplementary figures and images for: JAK3/STAT5 signaling‐triggered upregulation of PIK3CD contributes to gastric carcinoma development
Source: J Cell Commun Signal. 2024 Feb 7;18(1):e12017. doi: 10.1002/ccs3.12017 (PMC10964935; doi:10.1002/ccs3.12017)

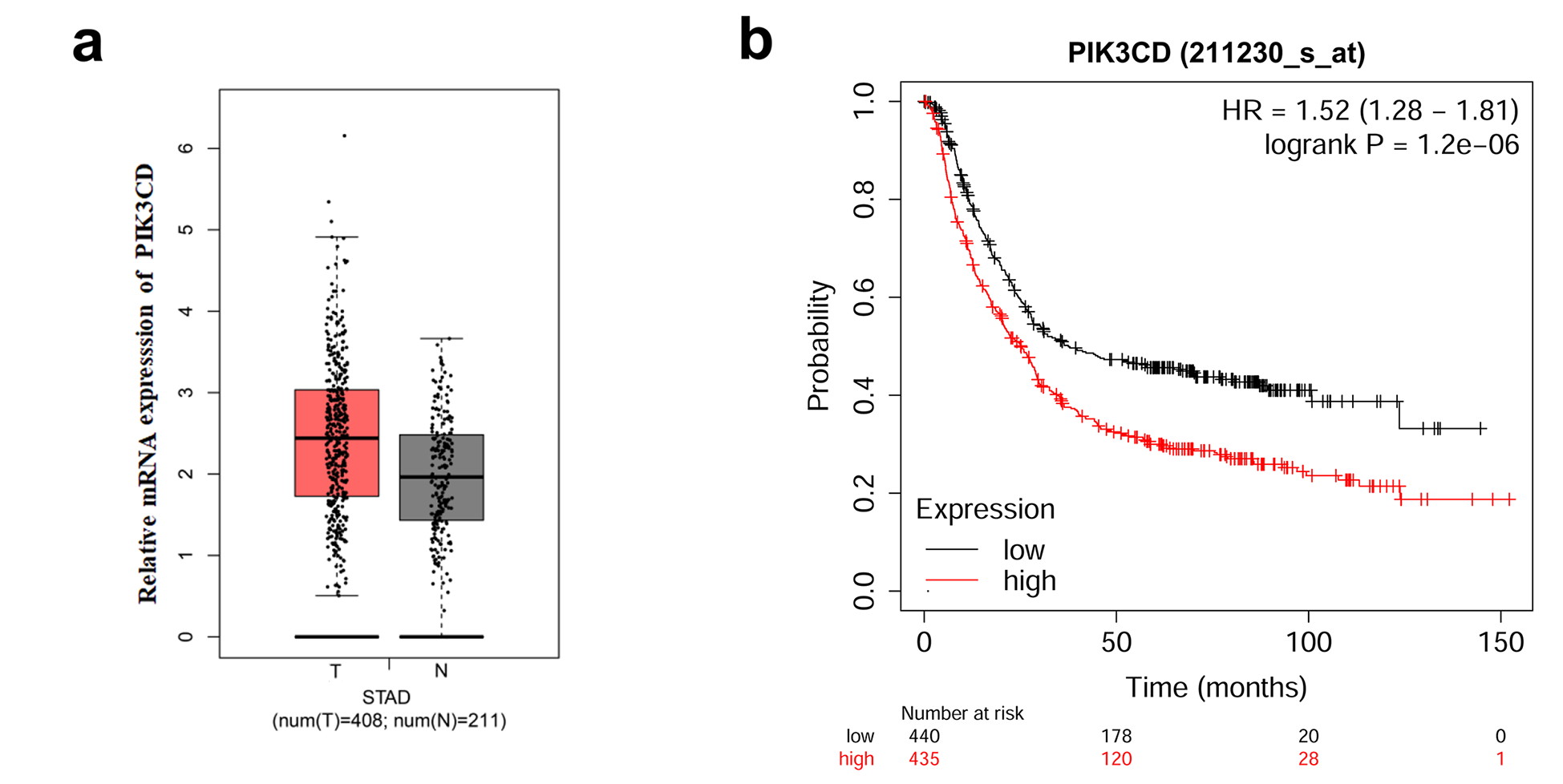

Supplement: Supplementary file 2 — Figure S1 [file CCS3-18-e12017-s002.tif]

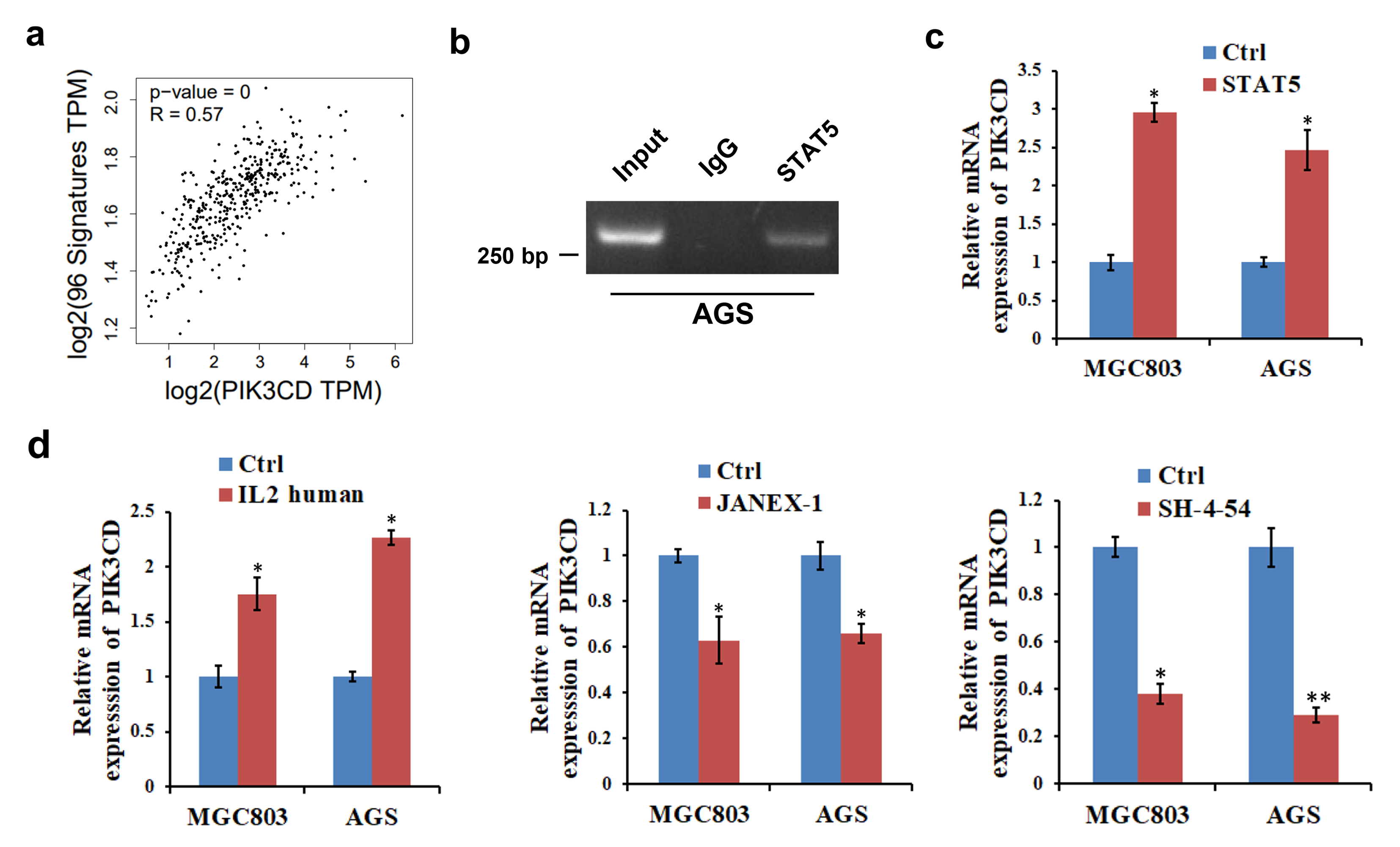

Supplement: Supplementary file 3 — Figure S2 [file CCS3-18-e12017-s003.tif]
